# Intravenous Arginine Administration Downregulates NLRP3 Inflammasome Activity and Attenuates Acute Kidney Injury in Mice with Polymicrobial Sepsis

**DOI:** 10.1155/2020/3201635

**Published:** 2020-05-11

**Authors:** Sharon Angela Tanuseputero, Ming-Tsan Lin, Sung-Ling Yeh, Chiu-Li Yeh

**Affiliations:** ^1^School of Nutrition and Health Sciences, College of Nutrition, Taipei Medical University, Taipei, Taiwan; ^2^Department of Surgery, National Taiwan University Hospital and College of Medicine, National Taiwan University, Taipei, Taiwan; ^3^Nutrition Research Center, Taipei Medical University Hospital, Taipei, Taiwan; ^4^Research Center of Geriatric Nutrition, College of Nutrition, Taipei Medical University, Taipei, Taiwan

## Abstract

Acute kidney injury (AKI) is a major complication of sepsis. Nucleotide-binding domain-like receptor protein 3 (NLRP3) inflammasomes are multiprotein complexes that mediate septic AKI. L-arginine (Arg) is a conditionally essential amino acid in catabolic conditions and a substrate for nitric oxide (NO) production; however, its use in sepsis is controversial. This study investigated the effect of intravenous Arg supplementation on modulating NLRP3 inflammasome activity in relation to septic AKI. Mice were divided into normal control (NC), sham, sepsis saline (SS), and sepsis Arg (SA) groups. In order to investigate the role of NO, L-N6-(1-iminoethyl)-lysine hydrochloride (L-NIL), an inducible NO synthase inhibitor, was administered to the sepsis groups. Sepsis was induced using cecal ligation and puncture (CLP). The SS and SA groups received saline or Arg via tail vein 1 h after CLP. Mice were sacrificed at 6, 12, and 24 h after sepsis. The results showed that compared to the NC group, septic mice had higher plasma kidney function parameters and lower Arg levels. Also, renal NLRP3 inflammasome protein expression and tubular injury score increased. After Arg treatment, plasma Arg and NO levels increased, kidney function improved, and expressions of renal NLRP3 inflammasome-related proteins were downregulated. Changes in plasma NO and renal NLRP3 inflammasome-related protein expression were abrogated when L-NIL was given to the Arg sepsis groups. Arg plus L-NIL administration also attenuated kidney injury after CLP. The findings suggest that intravenous Arg supplementation immediately after sepsis restores plasma Arg levels and is beneficial for attenuating septic AKI, partly via NO-mediated NLRP3 inflammasome inhibition.

## 1. Introduction

Sepsis is a life-threatening organ dysfunction syndrome due to dysregulated host responses to infection [1]. Among others, the kidneys are one of the first organs to be affected by sepsis since the kidneys receive 20% of the blood flow output, processing 120~150 mL of plasma each minute, and thus have high exposure to secreted proinflammatory mediators [2]. It was reported that 40%~50% of septic patients develop acute kidney injury (AKI) and thereafter have 6~8-fold higher mortality compared to those without AKI [3]. The pathophysiology of septic AKI is complex and multifactorial. Previous studies showed that deranged immune cell activation and proinflammatory cytokine production are the main causes of AKI. Insults from both infection and cell damages trigger persistent cycle of inflammatory response, in which innate immunity plays a major role [2, 4].

Inflammatory response occurs in almost all kinds of kidney diseases. Inflammasomes are protein complexes that form within activated immune cells and tissue-resident cells that lead to a series of inflammatory reactions [5]. NLRP3 is a member of the nucleotide-binding and oligomerization domain- (NOD-) like receptor family and was described as the inflammasome sensor [6]. After recognition of infecting microbials and cellular damage in a two-step mechanism, NLRP3 will form an activated complex with apoptosis-associated speck-like protein (ASC) and procaspase-1 which will subsequently cleave into IL-1*β* [7]. NLRP3 inflammasome responses to varieties of pathogens. The activation of NLRP3 inflammasome has been proved to contribute to the inflammatory response of sepsis-induced AKI, which causes an impaired kidney morphology, increased renal tubular cell apoptosis, and NLRP3-dependent proinflammatory cytokine (i.e., IL-1*β* and IL-18) production [8–10].

Arginine (Arg) is a nonessential amino acid that serves as the precursor of various metabolites and is the sole substrate of nitric oxide (NO) [11]. *De novo* synthesis of Arg is regulated by the kidneys [12]. Regarding the notion that sepsis is an Arg-deficient state [13], Arg supplementation was proposed and shown to have favorable effects in critically ill surgical patients [14, 15]. Also, Arg enhanced the immune response and protein turnover and showed beneficial effects in a porcine model of endotoxemia [16]. A study performed by our laboratory showed that intravenous Arg administration attenuated sepsis-induced lung injury [17]. Since NO is an inhibitor of caspase-1 [18], availability of NO may inhibit NLRP3 inflammasome activation and subsequent IL-1*β* and IL-18 production. We hypothesized that intravenous Arg administration may downregulate renal NLRP3 expression, possibly via NO signaling, and thus attenuate septic AKI. In order to clarify the role of NO in regulating the NLRP3 inflammasome associated with AKI, a specific inducible NO synthase (iNOS) inhibitor was administered in addition to Arg in a mouse model of polymicrobial sepsis in this study.

## 2. Materials and Methods

### 2.1. Animals

Male C57BL/6J mice (5 to 6 weeks old, weighing 20~25 g) were used in the experiment. All mice were subjected to acclimatization in a temperature (21 ± 2°C) and humidity controlled room (50%~55%) with a 12 h light-dark cycle in the Laboratory Animal Center at Taipei Medical University (TMU), Taipei, Taiwan. During the period of study, all mice were given standard chow diet and water *ad libitum*. Care of laboratory animals was in full compliance with the Guide for the Care and Use of Laboratory Animals (National Research Council, 1996). Experimental protocols were approved by the TMU's Animal Care and Use Committee.

### 2.2. Study Protocol

Mice were randomly assigned to a normal control (NC) group (*n* = 6), a sham group (*n* = 6), a septic saline (SS, *n* = 24) group, and a septic Arg (SA, *n* = 24) group. Polymicrobial peritonitis sepsis was induced by cecal ligation and puncture (CLP) as described previously [17]. Mice were anesthetized, a midline incision (1~1.5 cm) was made in the abdominal wall, and the cecum was identified. The cecum was exposed, and approximately 50% of the distal end of the cecum was ligated with 3-0 silk. Using a 23-gauge needle, it was punctured twice on the cecal end, gently compressed to extrude a small amount of feces, and then replaced back in the abdomen. The incision was closed in two layers using 2-0 silk sutures. The animals were resuscitated with subcutaneous sterile saline (40 mL/kg body weight (BW)) after the CLP operation. Mice in the sham group were subjected to the same surgical procedure except for CLP. After surgery, animals were allowed free access to food and water. All CLP manipulations were performed by the same person to ensure consistency. One hour after CLP, mice were intravenously injected with a bolus (100 *μ*L) of saline or Arg solution (300 mg/kg BW) via a tail vein. This dosage of Arg was previously proven to have beneficial effects in resolving inflammatory responses in a catabolic condition [19]. Mice were sacrificed at 6, 12, and 24 h after CLP to investigate the dynamic inflammatory responses. Mice were anesthetized with an intraperitoneal (IP) injection of Zoletil® (Virbac, Carros, France; 25 mg/kg BW) and Rompun (Bayer, Leverkusen, Germany; 10 mg/kg BW), and blood samples were collected by cardiac puncture. Blood samples from mice were collected into tubes containing heparin and were centrifuged at 1500 × g at 4°C for 15 min to collect plasma which was stored in -80°C for further analysis. The upper half of a kidney was separated for histological analysis, while the remaining part was kept at -80°C for further analysis. To investigate the role of NO, L-N (6)-iminoethyl-lysine (L-NIL) (Sigma, St. Louis, MO, USA), an inducible (i) NOS inhibitor, was administered to mice in the septic saline (SSL, *n* = 15) and septic Arg (SAL, *n* = 15) groups. L-NIL (3 mg/kg BW) was given intraperitoneally at the end of CLP and at 6 h after sepsis induction [20]. Mice in the SSL and SAL groups were sacrificed at 6, 12, and 24 h to collect blood and kidney samples. Survival rates were expressed as the number of mice which survived until the designated sacrifice time point per total amount of mice.

### 2.3. Analysis of Plasma Amino Acid Concentrations

Plasma samples were prepared using a Waters AccQTag derivatization kit (Manchester, UK) and subjected to ultraperformance liquid chromatography (UPLC) separation using the ACQUITY UPLC system (Waters). A multiple reaction monitoring (MRM) analysis was performed using a Xevo TQ-XS (Waters) mass spectrometer. Data were analyzed using Waters MassLynx 4.2 software and quantified using TargetLynx.

### 2.4. Analysis of Plasma NO Concentrations

Plasma NO was measured using a Total Nitric Oxide and Nitrate/Nitrite Assay (R&D Systems, Minneapolis, MN, USA) according to the manufacturer's instructions. Briefly, the amount of NO was calculated based on the enzymatic conversion of nitrate to nitrite by nitrate reductase, followed by the Griess reaction which produces a chromophoric compound detectable at wavelengths of 540/570 nm. The difference in measurements of nitrite and nitrate was considered the concentration of NO.

### 2.5. Analysis of Plasma Biomarkers for Kidney Function and Injury

Plasma creatinine (Cr) and blood urea nitrogen (BUN) were sent for laboratory analysis in the National Laboratory Animal Center, Taipei, Taiwan. Values are expressed in mg/dL. For measurement of plasma neutrophil gelatinase-associated lipocalin (NGAL), an indicator of AKI, 40 *μ*L of the obtained plasma was centrifuged at 11000 × g for 10 min at 4°C. Supernatants were measured by Quantikine® enzyme-linked immunosorbent assay (ELISA) kits (R&D Systems) according to the manufacturer's instructions, and results are expressed in *μ*g/mL.

### 2.6. Analysis of Renal Lipid Peroxidation Levels

Lipid peroxidation was analyzed based on thiobarbituric acid-reactive substance (TBARS) levels. One half of kidney tissues (0.05~0.07 g) twas homogenized in 250~350 *μ*L of T-PER™ tissue protein extraction reagent (Thermo, Rockford, IL, USA) and centrifuged to obtain lysates. Protein lysates were supplemented with 0.22% H_2_SO_4_, 0.67% thiobarbituric acid, and 10% phosphotungstic acid, boiled at 95°C, extracted with 1-butanol (Sigma), and centrifuged at 700 × g for 15 min at 4°C. The upper layer was collected and analyzed fluorometrically at 555/515 nm. Values were expressed as *μ*M malondialdehyde (MDA)/*μ*g protein.

### 2.7. Renal NLRP3 Inflammasome-Related Protein Expressions

Protein expressions of NLRP3 inflammasome-related species were analyzed by Western blotting. Briefly, kidney protein extracts were separated by 8%~15% sodium dodecyl sulfate polyacrylamide gel electrophoresis (SDS-PAGE), transferred to polyvinylidene difluoride membranes, blocked, and probed with primary antibodies such as anti-NLRP3, IL-1*β*, ASC (Cell Signaling Technology, Danvers, MA), and caspase-1 (Abcam, Cambridge, UK) overnight at 4°C. After incubation with the secondary antibody, proteins were visualized in a chemiluminescent solution (Merck Millipore, Burlington, MA, USA) using the BioSpectrum Imaging System (UVP, Upland, CA, USA) and quantified using Image-Pro Plus software version 4.5 (Media Cybernetics, Silver Springs, MD, USA). Densities of target proteins were normalized against *β*-actin.

### 2.8. Kidney Histology

The middle segments of kidney tissues were collected and fixed with 4% paraformaldehyde. Series of 5 *μ*m thick sections stained with hematoxylin and eosin (H&E) were examined to determine the morphology of the kidneys. Digital images at 200x magnification per section were captured. Five fields per section were analyzed for morphological lesions. Images were assessed by Image-Pro Plus software, and a scoring system based on Kuruş et al. [21] was used as follows: 0 indicates no tubular injury; 1 indicates <10% of tubules injured; 2 indicates 10%~25% of tubules injured; 3 indicates 26%~50% of tubules injured; 4 indicates 51%~75% of tubules injured; and 5 indicates >75% of tubules injured.

### 2.9. Statistical Analysis

Data are presented as the mean ± standard deviation (SD). Results were analyzed using GraphPad Prism 5 software (San Diego, CA, USA). Differences between groups were assessed using a one-way analysis of variance (ANOVA) followed by Tukey's post hoc test. Values were considered statistically significant at *P* < 0.05.

## 3. Results

### 3.1. BW Change and Survival Rates

There were no differences in initial BWs before the sham or CLP operation (data not shown). No difference in survival rates was observed between the two septic groups at 24 h after CLP (66% and 72% in both the SS and SA groups).

### 3.2. Changes in Plasma Amino Acid Levels

**Figure 1:**
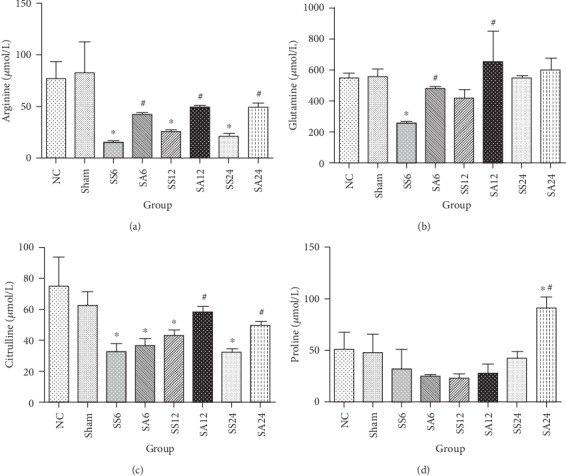
Plasma arginine, glutamine, citrulline, and proline concentrations of the normal and experimental groups. NC: normal control group; Sham: sham group; SS: sepsis group with saline injection sacrificed at 6, 12, and 24 h after cecal ligation and puncture (CLP); SA: sepsis group with arginine injection sacrificed at 6, 12, and 24 h after CLP. Results are presented as the mean ± SD; *n* = 6 for each group. Differences between groups were analyzed with a one-way ANOVA with Tukey's post hoc test. ^∗^Significantly differs from the NC group; ^#^significantly differs from the SS groups at the same time point (*P* < 0.05).

Arg concentrations had significantly decreased by 6 h and sustained the levels till the 24 h time point after sepsis compared to the NC and sham groups. Other amino acids, such as glutamine and citrulline, were also depleted in the septic groups at 6 h or the all time points. After Arg administration, levels of Arg had significantly increased at all time points, while glutamine exclusively increased at 6 and 12 h compared to the saline-treated groups. Citrulline and proline concentrations had significantly increased after 12 and/or 24 h post-CLP (Figure 1).

### 3.3. Plasma Biomarkers of Kidney Function

NGAL had significantly increased at 24 h of sepsis compared to levels in the NC and sham groups. Levels of Cr and BUN were higher in the sepsis groups than those in the NC and sham groups at 12 and 24 h. Levels of NGAL significantly dropped by 24 h, while Cr and BUN levels had decreased at 12 and 24 h in the Arg-treated sepsis group (SA group) when compared to the corresponding SS group (Table 1).

### 3.4. Plasma NO Levels with or without an iNOS Inhibitor

**Figure 2:**
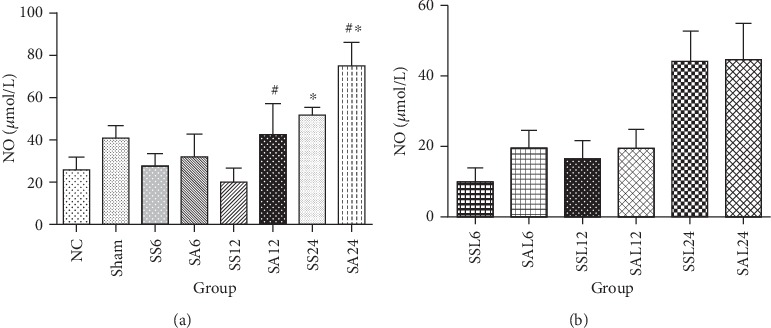
Plasma nitric oxide (NO) levels in (a) the normal and experimental groups. NC: normal control group; Sham: sham group; SS: sepsis group with saline injection sacrificed at 6, 12, and 24 h after cecal ligation and puncture (CLP); SA: sepsis group with arginine injection sacrificed at 6, 12, and 24 h after CLP. (b) Experimental groups with the inducible NO synthase (iNOS) inhibitor, L-N (6)-iminoethyl-lysine (L-NIL) administration. SSL: sepsis group with saline plus L-NIL, sacrificed at 6, 12, and 24 h after CLP; SAL: sepsis group with Arg plus L-NIL, sacrificed at 6, 12, and 24 h after CLP. Results are presented as the mean ± SD; *n* = 6 for each group. Differences between groups were analyzed using a one-way ANOVA with Tukey's post hoc test. ^∗^Significantly differs from the NC group; ^#^significantly differs from the SS groups at the same time point in (a).

There were no differences in NO levels among the NC, sham, and sepsis groups at 6 or 12 h after CLP. Compared to the NC group, NO production in the septic groups had increased by 24 h. Arg-treated groups had higher NO levels than the saline groups at 12 and 24 h post-CLP (Figure 2(a)). In the sepsis groups treated with L-NIL, no differences in plasma NO concentrations were observed among the NC, sham, and sepsis groups at the various time points (Figure 2(b)).

### 3.5. Lipid Peroxide Levels in the Kidneys

**Figure 3:**
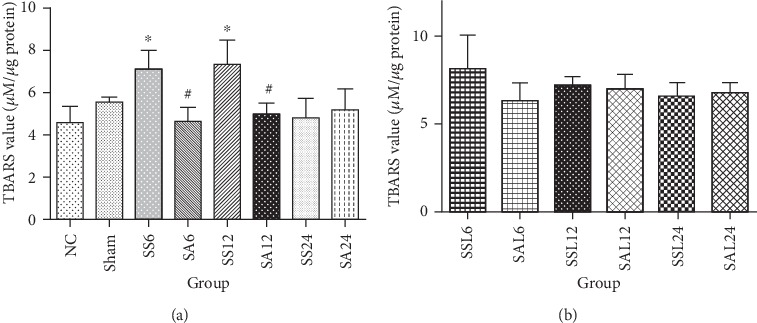
Renal thiobarbituric acid-reactive substance (TBARS) values in (a) the normal and experimental groups. NC: normal control group; Sham: sham group; SS: sepsis group with saline injection sacrificed at 6, 12, and 24 h after cecal ligation and puncture (CLP); SA: sepsis group with arginine injection sacrificed at 6, 12, and 24 h after CLP. (b) Experimental groups with the nitric oxide synthase (iNOS) inhibitor, L-N (6)-iminoethyl-lysine (L-NIL) administration. SSL: sepsis group with saline plus L-NIL, sacrificed at 6, 12, and 24 h after CLP; SAL: sepsis group with Arg plus L-NIL, sacrificed at 6, 12, and 24 h after CLP. Results are presented as the mean ± SD; *n* = 6 for each group. Differences among groups were analyzed using a one-way ANOVA with Tukey's post hoc test. ^∗^Significantly differs from the NC group; ^#^significantly differs from the SS groups at the same time point (*P* < 0.05) in (a).

TBARS values in the sepsis groups had significantly increased at 6 and 12 h after CLP compared to the NC group. After Arg treatment, TBARS values at 6 and 12 h post-CLP were significantly lower compared to the respective saline group (Figure 3(a)). In the sepsis groups treated with L-NIL, no differences in TBARS concentrations were observed between the saline- and Arg-treated sepsis groups (Figure 3(b)).

### 3.6. Kidney NLRP3 Inflammasome-Associated Protein Expression in Sepsis Groups

**Figure 4:**
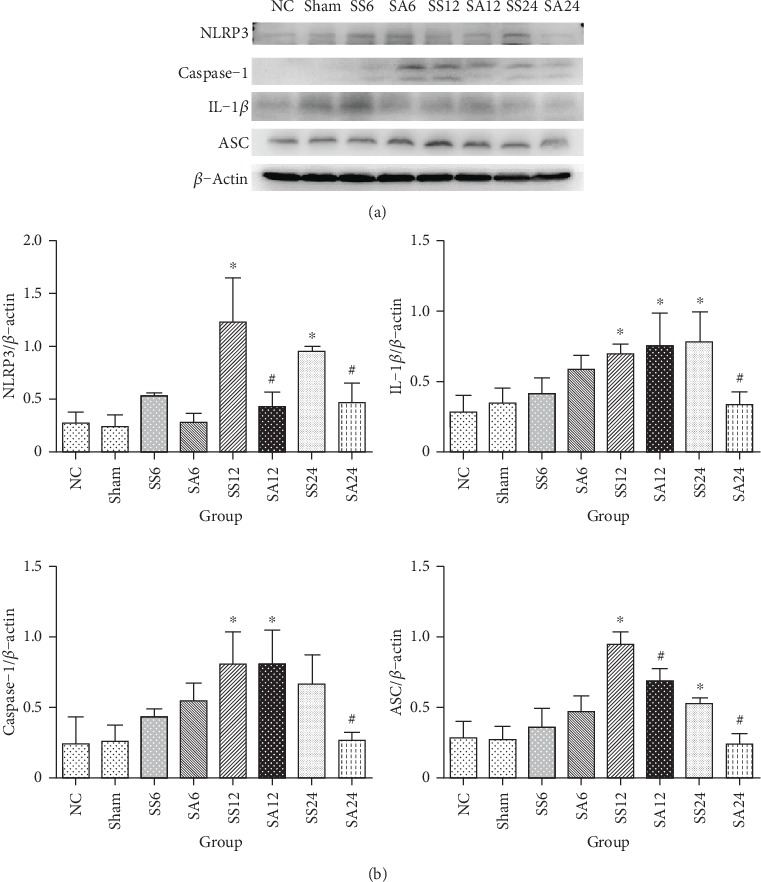
Protein levels of the nucleotide-binding domain-like receptor protein 3 (NLRP3) inflammasome complex in renal tissues. (a) Protein expressions of NLRP3, apoptosis-associated speck-like protein containing CARD (ASC), interleukin (IL)-1*β*, and caspase-1. Whole-tissue lysates were analyzed by immunoblotting, and *β*-actin was used as a loading control. (b) Densitometric analysis of blots corrected by the protein loading control. NC: normal control group; Sham: sham group; SS: sepsis group with saline injection sacrificed at 6, 12, and 24 h after cecal ligation and puncture (CLP); SA: sepsis group with arginine injection sacrificed at 6, 12, and 24 h after CLP. Results of the densitometric analysis are presented as the mean ± SD; *n* = 6 for each group. Differences among groups were analyzed using a one-way ANOVA with Tukey's post hoc test. ^∗^Significantly differs from the NC group; ^#^significantly differs from SS groups at the same time point (*P* < 0.05).

Compared to the NC and sham groups, protein levels of caspase-1 had increased by 12 h while NLRP3, ASC, and IL-1*β* had increased by both 12 and 24 h after CLP. After Arg treatment, expressions of NLRP3 and ASC were significantly reduced at 12 and 24 h, while caspase-1 and IL-1*β* exhibited reduced expression at 24 h (Figure 4).

### 3.7. Kidney NLRP3 Inflammasome-Associated Protein Expressions in Sepsis Groups with the iNOS Inhibitor

**Figure 5:**
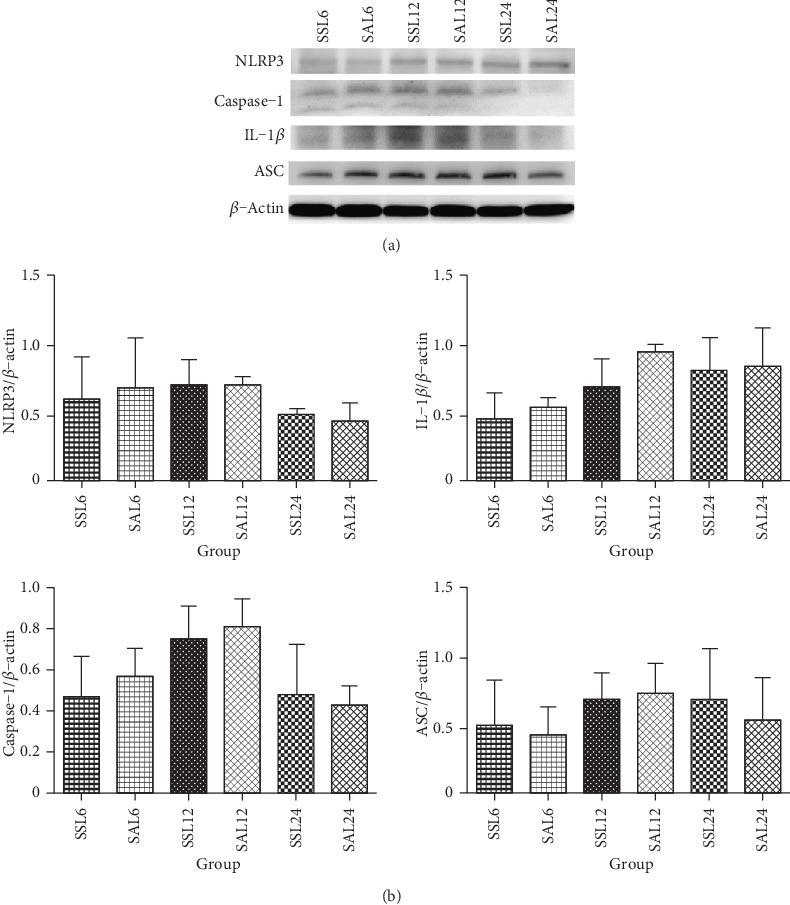
Kidney nucleotide-binding domain-like receptor protein 3 (NLRP3) inflammasome-associated protein expressions in groups treated with the nitric oxide synthase (iNOS) inhibitor, L-N (6)-iminoethyl-lysine (L-NIL). (a) Protein expressions of NLRP3, apoptosis-associated speck-like protein containing CARD (ASC), interleukin- (IL-) 1*β*, and caspase-1. Whole-tissue lysates were analyzed by immunoblotting, and *β*-actin was used as a loading control. (b) Densitometric analysis of blots corrected by the protein loading control. SSL: sepsis group with saline plus L-NIL, sacrificed at 6, 12, and 24 h after CLP; SAL: sepsis group with Arg plus L-NIL, sacrificed at 6, 12, and 24 h after CLP. Results of the densitometric analysis are presented as the mean ± SD; *n* = 6 for each group. Differences among group were analyzed using a one-way ANOVA with Tukey's post hoc test.

There were no differences in NLRP3, ASC, caspase-1, and IL-1*β* protein levels between the saline- and Arg-treated groups at each time point (Figure 5).

### 3.8. Kidney Histology of the Sepsis Groups with or without the iNOS Inhibitor

**Figure 6:**
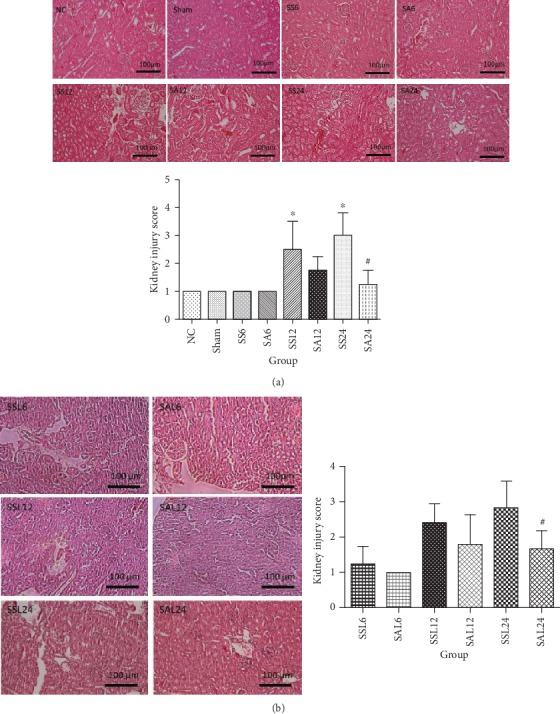
Histology and quantification of kidney tissues. Images were assessed using Image-Pro Plus software, and representative histological images are shown at 200x magnification. Microphotographs and semiquantification of H&E staining in (a). NC: normal control group; Sham: sham group; SS: sepsis group with a saline injection sacrificed at 6, 12, and 24 h after cecal ligation and puncture (CLP); SA: sepsis group with an arginine injection sacrificed at 6, 12, and 24 h after CLP; (b) SSL: sepsis group with saline plus L-NIL, sacrificed at 6, 12, and 24 h after CLP; SAL: sepsis group with Arg plus L-NIL, sacrificed at 6, 12, and 24 h after CLP. Results are presented as the mean ± SD; *n* = 6 for each group. Differences between groups were analyzed using a one-way ANOVA with Tukey's post hoc test. ^∗^Significantly differs from the NC group (*P* < 0.05); ^#^significantly differs from the SS groups at the same time point (*P* < 0.05).

Tubular injury was observed in the sepsis groups at 12 and 24 h, as indicated by vacuole formation and sloughing of tubular epithelial cells. In contrast, Arg-treated groups had significantly lower injury scores compared to the SS groups at 24 h (Figure 6(a)). Despite being treated with L-NIL, less tubular damage accompanied by a lower injury score was also observed in the Arg sepsis group at 24 h after CLP (Figure 6(b)).

## 4. Discussion

There is controversy surrounding the supplementation of Arg in the critically ill, especially in septic patients. An investigation by Bertolini et al. [22] found that mortality rates increased in the septic patients with Arg treatment. However, some clinical studies found that an infusion with Arg did not result in any adverse changes in hemodynamic parameters [23, 24]. Supplemental Arg by either an enteral or parenteral route is safe and may be beneficial to septic patients [23]. Sepsis is considered as an Arg-deficient state [13]. The drop in Arg availability in sepsis may be due to increased demand of amino acid for protein synthesis [12] or the activation of myeloid-derived suppressor cells which may enhance the arginase activity [25]. In a rodent model of sepsis, arterial levels of Arg were reduced at as early as 90 min after the onset of LPS induction [26]. Therefore, in this study, Arg was injected immediately after CLP induction to account for the expected decrease in plasma Arg. Findings from the current study showed that Arg administration enhanced NO production and downregulated NLRP3 inflammasome-related protein expression that may consequently result in attenuating septic AKI.

The finding of this study showed that NO levels after sepsis induction did not significantly increase until 24 h, while Arg-treated groups showed significantly higher NO levels since 12 h onwards. Poeze et al. [27] observed that in the porcine endotoxemia model, plasma NO levels increased significantly in the LPS-infused animals pretreated with Arg, while there was no difference in NO of the untreated animals. This phenomenon may be due to the compartmentalization of NO, where some organs could be deprived of NO even when the plasma levels are not changed [28]. Moreover, sepsis also induces the activation of arginase. The competition between arginase and NOS for using Arg as a substrate may delay the elevation of plasma NO that may explain the late response of increased NO in the saline sepsis group. iNOS has been known to contribute to excessive, long-lasting production of NO which is possibly responsible for hypotension and shock [28]. Excessive iNO production is considered as a source of reactive nitrogen species (RNS). Paradoxically, depletion of Arg also enhances the iNOS-derived formation of O_2_^−^ due to iNOS uncoupling reaction [29]. Increased Arg availability also inhibits the production of O_2_^−^ [30]. A previous in vitro study showed that decreasing concentrations of Arg enhanced iNOS-induced ROS/RNS production in cardiac myocytes [31]. In this study, Arg supplementation increased the availability of Arg to be converted to NO and may consequently prevent O_2_^−^ and peroxynitrite formation in current sepsis condition.

In respect to kidney function, NGAL reflects activated neutrophils during innate immune activation [32] and is a more-specific marker of tubular injury compared to kidney function parameters such as Cr and BUN [33]. A previous study reported that rats with Arg-supplemented diets had lower NGAL values after a uninephrectomy and kidney stone induction [34]. Significant reductions in plasma Cr, BUN, and NGAL levels observed in the Arg sepsis group compared to the saline sepsis group are suggestive of attenuated renal injury following Arg treatment.

Previous studies showed that resident mononuclear cells, such as macrophage and dendritic cells, express all parts of the NLRP3 inflammasome (NLRP3, ASC, and procaspase-1) in kidney tissues, but the expression levels are low in normal condition [35–37]. In the septic AKI state, NLRP3 inflammasome expressed by resident mononuclear cells and the recruited leukocytes are upregulated to secrete mature inflammatory cytokines [38]. On the other hand, renal parenchymal cells such as renal tubular epithelial cells, podocytes, glomerular endothelial cells, and mesangial cells contain significant amount of NLRP3 that expressed under inflammatory conditions [39, 40]. In this study, we evaluated the role of NO on septic AKI because NO is one of the suppressors of NLRP3. A previous study showed that NO could suppress caspase-1 in murine macrophages, resulting in decreased IL-1*β* [41]. Macrophages treated with S-nitroso-N-acetylpenicillamine, an NO donor, showed lower NLRP3 activation and IL-1*β* production [42]. *In vivo*, mice treated with the NOS inhibitor, N*ω*-nitro-L-arginine methyl ester hydrochloride (L-NAME), showed significantly increased IL-1*β* [43]. In this study, we found that the expression of NLRP3-associated proteins, including NLRP3, ASC, caspase-1, and IL-1*β*, was significantly upregulated at 12 and 24 h after sepsis. Arg administration downregulated expressions of NLPR3 inflammasome-related proteins. However, the favorable effects were abrogated when Arg sepsis groups were treated with L-NIL. These findings suggest that Arg administration alleviates sepsis-induced renal NLRP3 inflammasome activation, and NO plays an important role in suppressing NLPR3 inflammasome expression.

A characteristic hallmark of septic AKI is tubular cell vacuolization and displacement of the nucleus to the periphery of the cell [44], which could be caused by increased ROS production in tubules with sluggish blood flow [2]. In this study, we found that sepsis-induced tubular cell damage was obvious, and the kidney injury score was elevated since 12 h after CLP. Although the iNOS inhibitor, L-NIL provided in this study, proved that the Arg/NO pathway participates in suppressing renal lipid peroxide production and NLPR3 inflammasome activation, kidney histological improvements were independent of NO-mediated regulation.

Regardless of whether or not L-NIL was administered, Arg supplementation decreased the severity of tubular damage at the late phase of sepsis. This result indicated that the NO-mediated suppression of NLPR3 inflammasome expression might only be one of the mechanisms responsible for attenuating septic AKI. It is possible that the benefits of Arg may be mediated through other process. Firstly, restored Arg levels needed for physiological demand may help to attenuate organ injury. Arg degradation occurs via multiple pathways which produce numerous metabolites with biological importance that participate in the pathogenesis of kidney and other diseases [45]. In addition to NO and citrulline synthesis via NOS, Arg is also a substrate for ornithine production through urea cycle, which can be converted by ornithine aminotransferase into pyrolline-5-carboxylase and subsequently proline. In this study, the findings showed that septic mice administered with Arg maintained plasma glutamine levels, reversed sepsis-induced Arg decrement, and increased proline levels after sepsis. Glutamine is a specific amino acid with immunomodulatory properties. Previous studies found that glutamine improved vascular function [46], elicited a more-balanced lymphocyte regulation, and thus reduced kidney injury in septic mice [47]. Proline is an essential component of collagen [48]. The amino acid profile presented here in the Arg sepsis group may provide favorable effects in attenuating damage to kidney tissues. Secondly, Arg replacement may improve organ perfusion by restoring constitutive endothelial NOS. A previous study found that heterogeneous peritubular flow led to hypoxia in the cortical areas and increased flow in the medulla [49]. Uneven microcirculatory flow in the kidney tubules is one of the causes that drives septic AKI [2]. Arg administration may promote NO production by constitutive endothelial NOS causing improved organ perfusion. A previous study also revealed that Arg administration after CLP enhanced the mobilization of proangiogenic cells, which may play important roles in resolving vascular endothelium inflammation and ameliorating remote organ injury in a septic condition [17]. Thirdly, the effect of Arg on leukocytes during sepsis may also play roles in attenuating organ inflammatory response. A previous study carried out by Wang et al. [50] demonstrated that Arg supplementation enhanced macrophage phagocytic activity and promoted bacterial clearance in septic rats. In an *in vitro* study performed by our laboratory, we found that Arg administration with comparable or higher than physiological levels reduced cellular adhesion molecule expression, decreased neutrophil transendothelial migration, and thus attenuated inflammatory response in abdominal surgical condition [51]. However, the exact mechanism through which Arg is involved in attenuating septic AKI requires further investigation. There was a limitation in this study. Since the inflammasome immunohistochemistry staining was not performed, the effects of Arg on the exact location of the inflammatory cells in kidney tissues cannot be displayed here and therefore needed to be elucidated.

In summary, this study showed that Arg administration immediately after sepsis increased plasma Arg and NO concentrations, Arg/NO-mediated regulation decreased lipid peroxide levels and downregulated NLRP3 inflammasome-associated protein expressions. Since Arg plus L-NIL administration also attenuated kidney injury after CLP, the favorable effect of Arg resulting from NO-mediated NLRP3 inflammasome inhibition may be partly responsible for attenuating septic AKI. The findings of this study provide basic information and imply that a single dose of Arg administration may have benefits in restoring Arg levels and alleviating remote kidney injury in abdominal surgical patients at risk of postoperative infectious complications.

## Figures and Tables

**Table 1:** Kidney injury marker levels and kidney function indicators.

	NGAL (*μ*g/mL)	Creatinine (mg/dL)	BUN (mg/dL)
NC	0.11 ± 0.01	0.17 ± 0.02	33.90 ± 1.31
Sham	0.47 ± 0.36	0.20 ± 0.04	36.60 ± 1.51
SS6	7.92 ± 3.29	0.17 ± 0.05	34.03 ± 2.19
SA6	8.07 ± 0.93	0.13 ± 0.02	38.38 ± 5.85
SS12	7.59 ± 0.97	0.67 ± 0.07^∗^	82.15 ± 4.05^∗^
SA12	14.03 ± 4.19	0.35 ± 0.09^#^	55.55 ± 2.19^#^
SS24	53.51 ± 27.78^∗^	0.62 ± 0.08^∗^	115 ± 19.46^∗^
SA24	18.61 ± 1.59^#^	0.42 ± 0.10^#^	31.93 ± 18.56^#^

The experimental groups consisted of NC: normal control group; Sham: sham group; SS: sepsis group with saline injection sacrificed at 6, 12, and 24 h after cecal ligation and puncture (CLP); SA: sepsis group with arginine injection sacrificed at 6, 12, and 24 h after CLP. BUN: blood urea nitrogen; NGAL: neutrophil gelatinase-associated lipocalin-2. Data were analyzed using a one-way ANOVA with Tukey's post hoc test and presented as the mean ± SD. ^∗^Significantly differs from the NC group; ^#^significantly differs from the SS groups at the same time point (*P* < 0.05).

## Data Availability

All data described in the manuscript are available from the first author upon request.
